# A Numerical Investigation of a Plasmonic Sensor Based on a Metal-Insulator-Metal Waveguide for Simultaneous Detection of Biological Analytes and Ambient Temperature

**DOI:** 10.3390/nano11102551

**Published:** 2021-09-29

**Authors:** Nikolay L. Kazanskiy, Svetlana N. Khonina, Muhammad A. Butt, Andrzej Kaźmierczak, Ryszard Piramidowicz

**Affiliations:** 1 Samara National Research University, 443086 Samara, Russia; kazanskiy@ipsiras.ru (N.L.K.); khonina@ipsiras.ru (S.N.K.); 2 Institute of RAS-Branch of the FSRC “Crystallography and Photonics” RAS, 443001 Samara, Russia; 3 Institute of Microelectronics and Optoelectronics, Warsaw University of Technology, Koszykowa 75, 00662 Warszawa, Poland; andrzej.kazmierczak@pw.edu.pl (A.K.); ryszard.piramidowicz@pw.edu.pl (R.P.)

**Keywords:** plasmonic sensor, metal-insulator-metal waveguide, temperature sensor, refractive index sensor, polydimethylsiloxane

## Abstract

A multipurpose plasmonic sensor design based on a metal-insulator-metal (MIM) waveguide is numerically investigated in this paper. The proposed design can be instantaneously employed for biosensing and temperature sensing applications. The sensor consists of two simple resonant cavities having a square and circular shape, with the side coupled to an MIM bus waveguide. For biosensing operation, the analytes can be injected into the square cavity while a thermo-optic polymer is deposited in the circular cavity, which provides a shift in resonance wavelength according to the variation in ambient temperature. Both sensing processes work independently. Each cavity provides a resonance dip at a distinct position in the transmission spectrum of the sensor, which does not obscure the analysis process. Such a simple configuration embedded in the single-chip can potentially provide a sensitivity of 700 nm/RIU and −0.35 nm/°C for biosensing and temperature sensing, respectively. Furthermore, the figure of merit (*FOM*) for the biosensing module and temperature sensing module is around 21.9 and 0.008, respectively. *FOM* is the ratio between the sensitivity of the device and width of the resonance dip. We suppose that the suggested sensor design can be valuable in twofold ways: (i) in the scenarios where the testing of the biological analytes should be conducted in a controlled temperature environment and (ii) for reducing the influence on ambient temperature fluctuations on refractometric measurements in real-time mode.

## 1. Introduction

Over the past few years, plasmonic-based devices have gathered significant interest thanks to their unique properties, enabling significant enhancement of the sensitivity of photonic sensors [1,2]. Continuous advancements in nanofabrication alter expectations of the role of metals in the development of new optical devices, based on plasmonic effects, like surface plasmon polaritons (SPPs). These are attractive for use in nanophotonics, biosensing, electronics, imaging, and many other disciplines [3]. SPPs are firmly connected to metal–dielectric interfaces, reaching a depth of around 10 nm in metal (skin depth) and often more than 100 nm in dielectric material. The metal-insulator-metal (MIM) waveguide system is one of the most widely utilized plasmonic-based nanostructures for the implementation in integrated photonic circuits [4,5]. Such waveguides are plasmonic structures of a simple design, with two metal claddings surrounding an insulator, able to confine SPP mode at the subwavelength level. So far, numerous optical devices developed with this waveguide configuration have been widely investigated, for applications such as biosensensing [6,7,8], temperature sensing [9,10], optical signal switching [11], demultiplexing [12], splitting [5], and filtering [13].

Plasmonic sensors are extremely attractive and in great demand owing to their small footprint and high sensing capabilities as compared with sensors based on other platforms such as silicon photonics or optical fiber [1,14]. Because of their ability to overcome the diffraction limit of light, SPP waveguide structures, particularly MIM waveguides, have attracted much attention, with expectations of realizing highly integrated optical circuits owing to their small footprint, ease of integration, and good balance between light localization and transmission loss [15]. A biosensing semiconductor nanowire refractive index sensor with a sensitivity of 235 nm/RIU has been demonstrated [16]. In ref. [17], an optical fiber refractive index sensor based on long-period fiber grating has been suggested. The maximum experimental sensitivity for quasi-TM ring resonators was 135 nm/RIU, according to Xu et al. [18]. The bulk sensitivity of 270 nm/RIU was displayed by adjusting the waveguide thickness [19].

**Figure 1:**
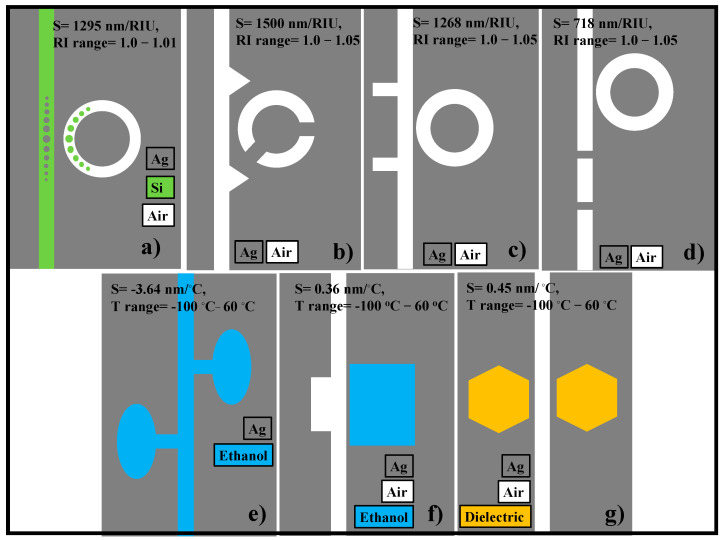
Schematic representation (top view) of single-purpose plasmonic sensor designs found in the literature. Refractive index sensor, (**a**) ring resonator coupled to an MIM waveguide containing tapered defects [24], (**b**) two triangle stubs coupled with a split-ring nanocavity [25], (**c**) two stubs and one ring resonator [26], and (**d**) two baffles and a coupled ring cavity [27]. Temperature sensors, (**e**) ethanol-sealed asymmetric ellipse resonators [9], (**f**) ethanol filled resonator cavity [23], and (**g**) dual laterally side-coupled hexagonal cavities [28].

Sensing is a hotspot among several interesting topics, and in the previous years, several plasmonic sensor designs based on MIM waveguides have been numerically investigated and proposed for refractive index and temperature sensing [20,21,22]. Although the sensitivity of these devices is relatively high, the proposed designs support only one application (either temperature or refractive index sensing) at a time. What is more, the manufacturing of these designs is challenging without leaving an error of a few nanometers owing to their additional complex geometric features. This may lead to a deterioration in the sensing performance. Our aim is not to criticize any previously developed sensor models; however, we would like to demonstrate that promising results can be achieved using a simple, multipurpose, and easy to implement sensor model. A few previously proposed complicated sensor designs used for refractive index and temperature sensing applications are shown in Figure 1a–g [9,23,24,25,26,27,28].

In ref. [24], the plasmonic sensor that combines a ring resonator containing circular tapered defects coupled to a MIM waveguide with tapered defects for refractive index sensing is proposed. The sensitivity of the device is around 1295 nm/RIU; however, the device design is so complicated that even a fabrication error of a few nanometers can deteriorate the device performance [24]. In ref. [25], another complicated refractive index sensor comprised of an MIM waveguide with symmetric two triangle stubs coupled to a circular split-ring resonator cavity is proposed. The sensitivity of the device is 1500 nm/RIU. However, for this sensor configuration, several parameters must be carefully optimized to obtain maximum sensing performance. A similar situation occurs with the designs proposed in ref. [26,27]. The numerical results presented in these studies seem to be attractive; however, the real challenge appears at the stage of fabrication of these devices where several variables must be optimized at a nanometer scale.

It has been recently demonstrated that the MIM plasmonic waveguide structures combined with thermal sensing media such as ethanol or PDMS can be efficiently utilized for temperature sensing applications [10,29,30,31]. Zhu et al. [9] proposed a sensor design that can only be used for temperature sensing and offers a very high sensitivity of −3.64 nm/°C. Furthermore, Zhu et al. numerically investigated a compact Fano resonance temperature sensor by utilizing polydimethylsiloxane (PDMS)-sealed semi-square ring resonator. PDMS is a mineral-organic polymer of the siloxane family. It is a polymeric organosilicon chemical that is commonly referred to as silicone. The silicone-based organic polymer PDMS is the most widely used. It is well-known for its distinct features—transparency over a wide wavelength range, the refractive index lower than in fused silica, good elasto-optic and thermo-optic coefficients, biocompatibility, and negligible absorption loss, to enumerate a few [32]. It also has a good mechanical property owing to low Young’s modulus; it is soft and deformable with no shrinkage. What is also important from the point of view of mass production is that the manufacturing process is cost-efficient and straightforward. Owing to the high thermo-optic coefficient of PDMS, the devices based on it are highly sensitive to temperature fluctuations. The sensor might be useful for temperature monitoring applications that need a high level of sensitivity. The sensitivity is around −4 nm/°C; however, the cavity shape is so complex that at least 5–6 parameters should be optimized to obtain the best sensing performance [10]. This restricts the flexibility in the fabrication process. Kong et al. proposed a temperature sensor utilizing ethanol in a resonant cavity providing a sensitivity of 0.36 nm/°C [23].

In diagnostic centers, the testing of the biological samples requires a controlled temperature environment for accurate results. Therefore, it is required to have a lab-on-chip solution that can examine the analytes and temperature at the same time. In this work, an attractive and straightforward sensor model is proposed that can be simultaneously employed as a biosensor and temperature sensor. The device contains two cavities, each of which is designated for a particular purpose. The resonance dips from both cavities are well separated, which makes evaluating their sensing performance extremely easy. The study is conducted via the 2D-finite element method (2D-FEM) by utilizing COMSOL Multiphysics software. The sensor design has no peculiar geometric features like stub [33] or nanodots [34], which can produce multiple resonance peaks/dips as well as add complications to the manufacturing process. Therefore, we believe that the proposed sensor design will be quite useful in realizing a multipurpose compact lab-on-chip sensor.

## 2. Single-Purpose Device Model and Simultaneous Parameters

In this section, the optimization of the geometric parameters of the square and circular cavity is carried out utilizing the 2D-FEM to determine the transmission spectrum of the device. The 2D model is preferred over the 3D one because it reduces the processing time and provides accurate results, as demonstrated in other works [24,34,35,36]. The geometry of the proposed sensor design is straightforward and does not involve any stub [19,27,37], nanodots [24,38], or other complex structures [36] that can complicate the fabrication process. The cavities are simple airholes that can be precisely etched with current fabrication technology [39].

**Figure 2:**
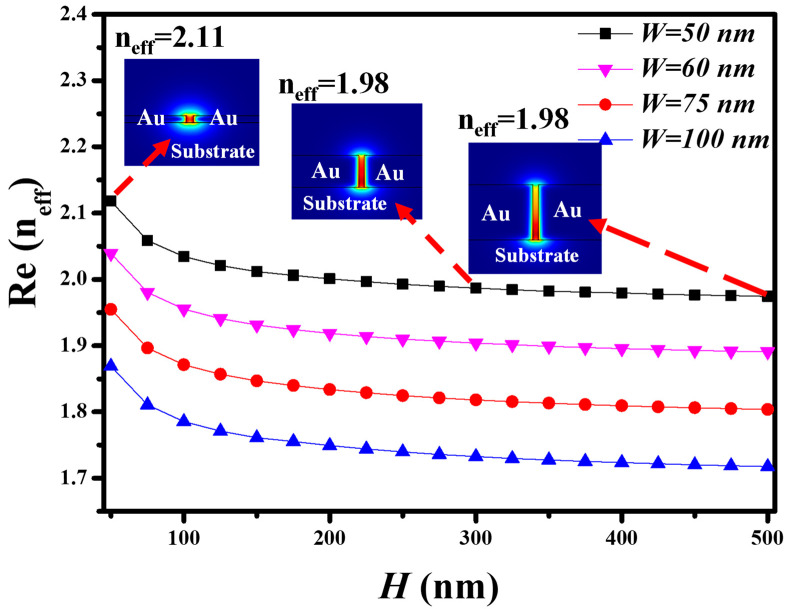
The real part of the effective refractive index versus the height (*H*) and slot width (*W*) of the MIM waveguide. Inset shows the cross-section E-field distribution in the MIM waveguide.

The real part of the effective refractive index (Re (n_eff_)) is plotted for the MIM waveguide heights (*H*) in the range of 50 nm to 500 nm at an operational wavelength of 1000 nm. The MIM waveguide is composed of gold (Au) and deposited on a quartz substrate. The waveguide widths of 50 nm, 60 nm, 75 nm, and 100 nm are also considered, as shown in Figure 2. In the case of *H* = 50 nm and *W* = 50 nm, Re (n_eff_) is at 2.11, which decreases and stabilizes to 1.98 as *H* approaches 300 nm and onward. The same trend follows for the MIM waveguide with *W* = 60 nm, 75 nm, and 100 nm. The inset of Figure 2 shows the H-field distribution at *H* = 50 nm, 300 nm, and 500 nm. Therefore, from this analysis, we can say that *H* = 300 nm or more can be used for the practical realization of the MIM waveguides.

**Figure 3:**
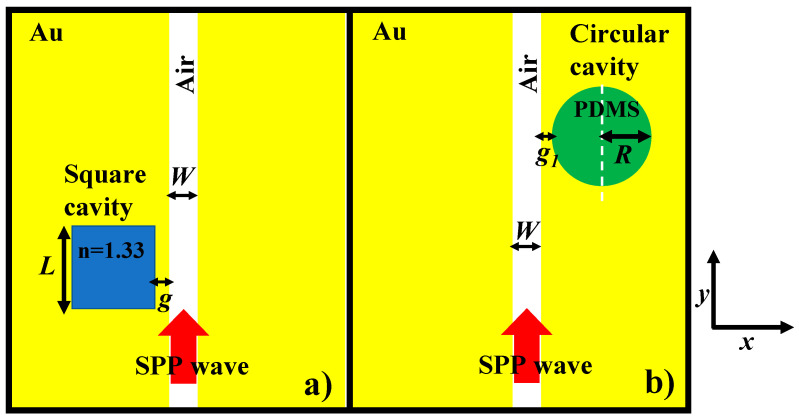
Schematic representation (top view) of the plasmonic cavity coupled to a bus waveguide: (**a**) square cavity filled with dielectric medium, *n* = 1.33, and (**b**) circular cavity filled with thermo-optic material, PDMS.

We aim to employ a square cavity and circular cavity on the same chip for biosensing and temperature sensing simultaneously. The device structure is composed of an Au layer deposited on a quartz substrate and the MIM waveguide configuration and cavities can be precisely created with the help of electron-beam lithography. However, at first, we would like to individually explore their spectral characteristics. The dielectric material of refractive index (n) = 1.33, which can be considered as water is filled in the square cavity and is side coupled to an MIM bus waveguide. The width of the MIM waveguide is denoted as *W,* which is fixed at 50 nm throughout this paper. The side length of the square cavity is represented as *L* and the gap between the bus MIM waveguide and the cavity is designated as *g*. Au is used as a plasmonic material because of its resistance to oxidation and biocompatibility. The relative dielectric constant of the Au is defined by the Drude–Lorentz dispersion model:

(1) $$\varepsilon =\varepsilon_{\infty }-\frac{\omega_{p}^{2}}{\omega^{2}+j\omega \gamma }$$

where $\varepsilon_{\infty }$ = 9.0685, $\omega_{p}$ = 135.44 × 10^14^ rad/s, and γ = 1.15 × 10^14^ rad/s [40]. The circular cavity is filled with PDMS material, which has a high thermo-optic coefficient. The cavity is separated from the bus waveguide with a small gap, which is represented as *g*_1_. The radius of the circular cavity is denoted as *R*. The fundamental mode that an MIM waveguide supports is an even mode with a transverse-magnetic (TM) polarization. As a result, an SPP mode is excited, which travels along the metal-dielectric boundary and couples to the designated cavity when the resonance condition is met [5,26,36,41,42]. The dispersion relation of the fundamental mode is defined as follows:

(2) $$\frac{\varepsilon_{i}p}{\varepsilon_{m}k}=\frac{1-e^{kW}}{1+e^{kW}},$$

(3) $$k=k_{o}\sqrt{{\left(\frac{\beta_{spp}}{k_{o}}\right)}^{2}-\varepsilon_{i}},\;p=k_{o}\sqrt{{\left(\frac{\beta_{spp}}{k_{o}}\right)}^{2}-\varepsilon_{m}},$$

(4) $$\beta_{spp}=n_{eff}k_{o}=n_{eff}\frac{2\pi }{\lambda },$$

where *W*, *λ*, *ε_i_,* and *ε_m_* denote the width of the bus waveguide, incident wavelength in vacuum, the relative dielectric, and metal permittivity, respectively. *n_eff_*, *β_spp_*, and *k_o_ = 2π/λ* are the effective refractive index, the propagation constant of SPPs, and the wave number, respectively. The schematic representation of the plasmonic sensors with a square cavity and a circular cavity is shown in Figure 3a,b, respectively.

**Figure 4:**
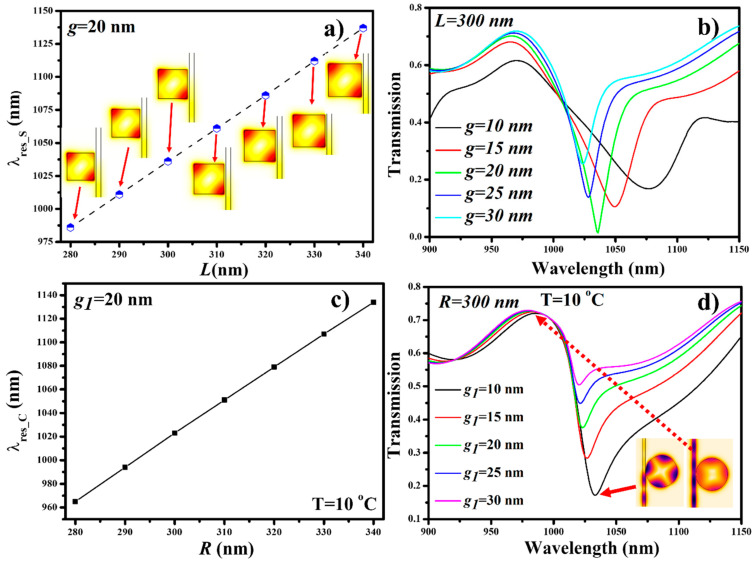
Spectral characteristics of square and circular cavity: (**a**) λ_res_S_ versus *L*, (**b**) effect of *g* on transmission, (**c**) λ_res_C_ versus *R*, and (**d**) effect of *g*_1_ on transmission.

The single-purpose device arrangement is so simple that only two variables should be optimized. For the square cavity, the influence of *L* on λ_res_S_ is determined at *g* = 20 nm, as shown in Figure 4a. It can be seen that λ_res_S_ performs a redshift as *L* increases from 280 nm to 340 nm, following a linear trend. The inset of Figure 4a shows the H-field distribution at the corresponding resonance wavelength. It is also important to determine the optimum coupling distance between the bus waveguide and the cavity to obtain the maximum coupling power (CP) and highest extinction ratio (ER). The transmission spectrum is plotted for the square cavity of *L* = 300 nm at *g* = 10 nm to 30 nm, as shown in Figure 4b. The maximum CP of >99% and highest ER of −18.6 dB are obtained for *g* = 20 nm.

For the circular cavity, *R* and *g*_1_ should be optimized for better CP and ER. The PDMS filled cavity is maintained at *g*_1_ = 20 nm and ambient temperature of 10 °C. The influence of *R* on λ_res_C_ is plotted in Figure 4c. Like a square cavity, λ_res_C_ performs a redshift as *R* increases from 280 nm to 340 nm in a linear fashion. Similarly, the coupling distance *g*_1_ is optimized by maintaining *R* = 300 nm, as shown in Figure 4d. The maximum CP and ER of >80% of −7.6 dB are obtained when *g*_1_ = 10 nm, respectively. The inset of Figure 4d shows the E-field distribution at on-resonance and off-resonance states when *g*_1_ = 10 nm.

## 3. Dual-Purpose Sensor Design

**Figure 5:**
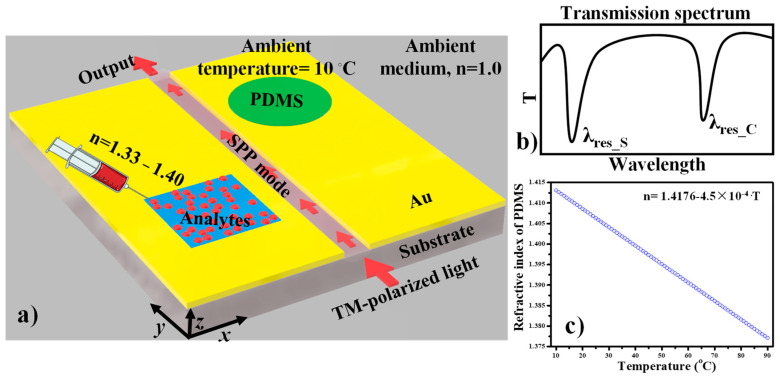
(**a**) Schematic representation (3D view) of a plasmonic sensor for simultaneous sensing of biological analytes and temperature, (**b**) The intended transmission spectrum of the proposed device, (**c**) Variation in the refractive index of the PDMS material versus the ambient temperature.

The schematic representation of the proposed sensor design for the simultaneous detection of biological analytes and temperature is shown in Figure 5a. The square cavity is filled with bio-analytes, whereas the circular cavity is filled with PDMS material. The variation in the refractive index of the PDMS layer concerning the temperature can be expressed as follows [43]:

n_PDMS_ (T) = 1.4176−4.5 × 10^−4^·T, (5)

where T is the ambient temperature. Both the cavities are at a distinct location and filled with dielectric material of different refractive indices which result in two separated resonance dips at unique positions in the spectrum (see Figure 5b). The first resonance dip is denoted as λ_res_S_, which corresponds to the square cavity. When the unknown analytes of refractive index higher than 1.33 are introduced into the cavity, λ_res_S_ performs a redshift owing to the variation in the effective refractive index, whereas λ_res_C_ is the second dip in the spectrum, which is connected to the circular cavity. The refractive index of the PDMS versus ambient temperature plot is shown in Figure 5c, which shows a linear relation between refractive index and temperature. The reduction in the refractive index of the PDMS layer due to the increase of the ambient temperature results in a blueshift of λ_res_C_. The operation of the sensor is based on the detection of a shift in resonance dips. It is worth noting that the change in λ_res_S_ does not affect λ_res_C_, and vice versa, because both the dips are well separated. This indicates that both the cavities work independently and any change in one cavity (in terms of refractive index) does not bring any change in the other. This feature encourages the realization of such devices that can act as biosensors and temperature sensors at the same time.

**Figure 6:**
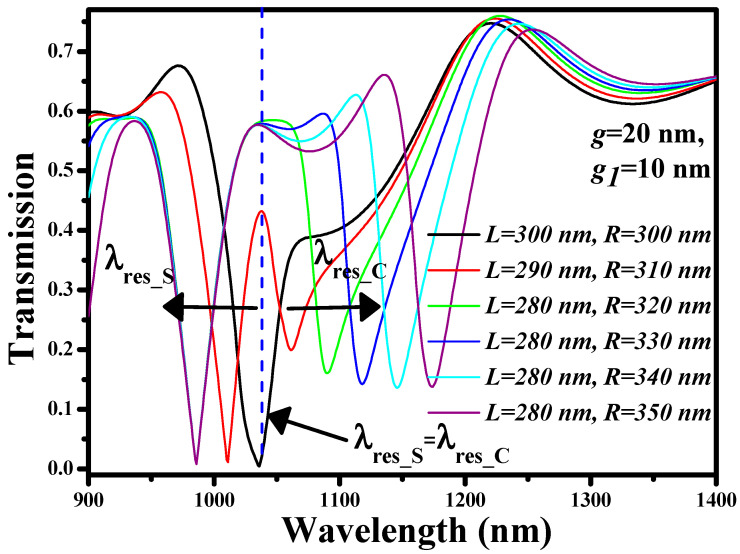
Transmission spectrum of square and circular cavity of various side lengths. The dotted blue line indicates the overlapping of λ_res_S_ and λ_res_C_.

The adequate separation between λ_res_S_ and λ_res_C_ must be sustained for the successful operation of the multi-purpose sensor. For that reason, we have plotted the transmission spectrum of the sensor for *L* = 280–300 nm and *R* = 300–350 nm, as shown in Figure 6. The remaining geometric parameters such as *g* and *g*_1_ are fixed at 20 nm and 10 nm, respectively. In the case when *L* = *R* = 300 nm, λ_res_S_ and λ_res_C_ overlap each other at 1036 nm, which is not a desirable situation for a multi-purpose sensing device. This is why an ample difference between the effective length of the cavities is crucial to attain two separate dips. For pronounced resonance dips, *L* and *R* are maintained at 280 nm and 340 nm, respectively, which provides λ_res_S_ = 985 nm and λ_res_C_ = 1145 nm, respectively. This, in turn, determines the free spectral range of 160 nm.

## 4. Modes of Operation, Results, and Discussion

**Figure 7:**
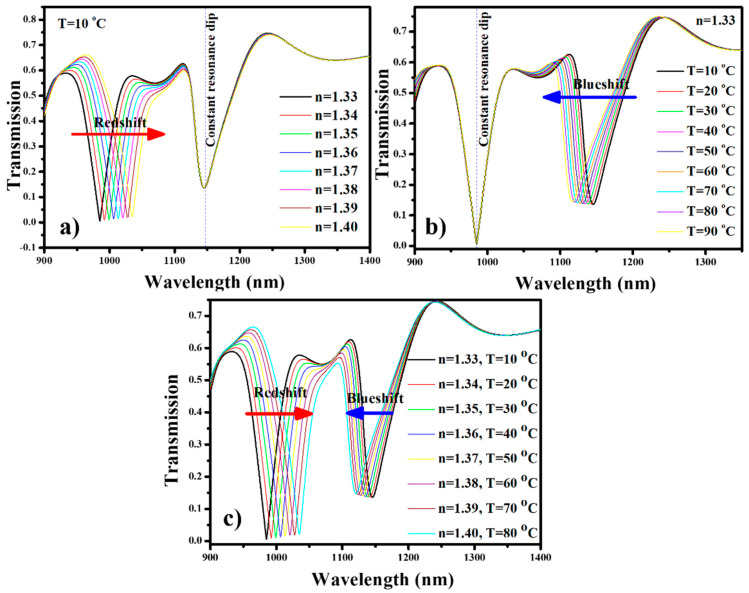
Transmission spectrum of the sensing device when employed as (**a**) biosensor, (**b**) temperature sensor, and (**c**) biosensor and temperature sensor.

The geometric parameters of the device are optimized in Section 3 and utilized for further sensing performance. Parameters such as *L*, *R*, *g*, *g*_1_, and *W* are maintained at 280 nm, 340 nm, 20 nm, 10 nm, and 50 nm, respectively. Here, we have considered three distinct scenarios in which the sensor can be employed. The first considered case is a biosensor. The square cavity is filled with a dielectric material (*n* = 1.33). As a result, λ_res_S_ = 985 nm is obtained. The ambient temperature is maintained at 10 °C; accordingly, the refractive index of PDMS material is preserved at 1.4131, which give rise to λ_res_C_ at 1145 nm. The refractive index of the analytes is varied between 1.33 and 1.40, which brings a redshift in λ_res_S_, as shown in Figure 7a. It is worth noting that λ_res_C_ is unaffected throughout this sensing process.

In the second case, the proposed device is employed as a temperature sensor and the square cavity is filled with a dielectric material (*n* = 1.33). The analysis is performed for the temperature range of 10–90 °C. As the ambient temperature increases, the refractive index of the PDMS material decreases, which brings a change in the effective refractive index of the SPP mode. Consequently, a blueshift is observed in λ_res_C_, as shown in Figure 7b. Once again, one can see that there is no shift in the position of λ_res_S_. This confirms that the proposed device can be employed for multipurpose sensing applications.

In the third case, the device is employed for the simultaneous detection of analytes and temperature. The refractive index of the analytes and ambient temperature are varied at the same time from 1.33 to 1.40 and 10 to 90 °C, respectively. As a result, λ_res_S_ and λ_res_C_ perform a redshift and blueshift, respectively. Both the resonance dip spectrums are well separated, which does not create any ambiguity in determining the shift in the resonance wavelength, as shown in Figure 7c.

**Figure 8:**
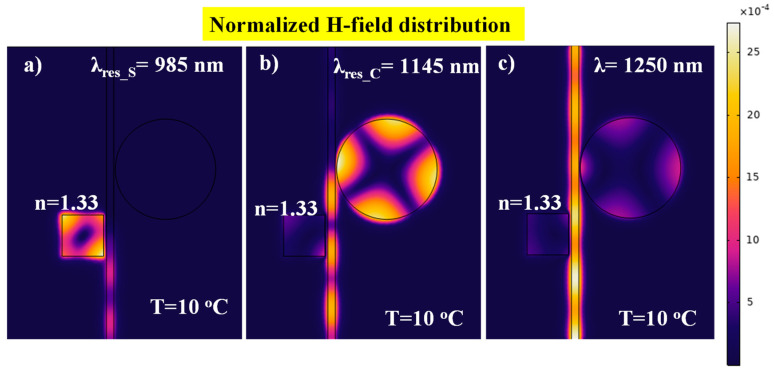
Normalized H-field distribution at (**a**) λ_res_S_ = 985 nm; (**b**) λ_res_S_ = 1145 nm; and (**c**) non-resonant mode, λ = 1250 nm. The geometric parameters used in this analysis are *W* = 50 nm, *L*= 280 nm, *R* = 340 nm, *g* = 20 nm, and *g*_1_ = 10 nm.

The normalized H-field distributions corresponding to the case when resonance occurs at one of sensors and to the case where no resonance occurs are plotted in Figure 8. In the first case of the square resonator, acting as a biosensor, the SPP mode is confined in the cavity at the resonance wavelength λ_res_S_ = 985 nm, as shown in Figure 8a. This resonance dip of the transmission spectrum must be traced while the biological samples will be analyzed with this sensor. In the second case, the H-field distribution at λ_res_S_ = 1145 nm is plotted, which shows the confinement of SPP mode in the circular cavity, as shown in Figure 8b. As the temperature of the ambient medium increases, the refractive index of the PDMS layer varies, resulting in a blueshift of the resonant wavelength λ_res_C_. For temperature sensing applications, a shift in λ_res_C_ must be tracked. In the third case, the H-field is plotted at λ = 1250 nm, which corresponds with the non-resonant transmission of both resonators, as shown in Figure 8c.

**Figure 9:**
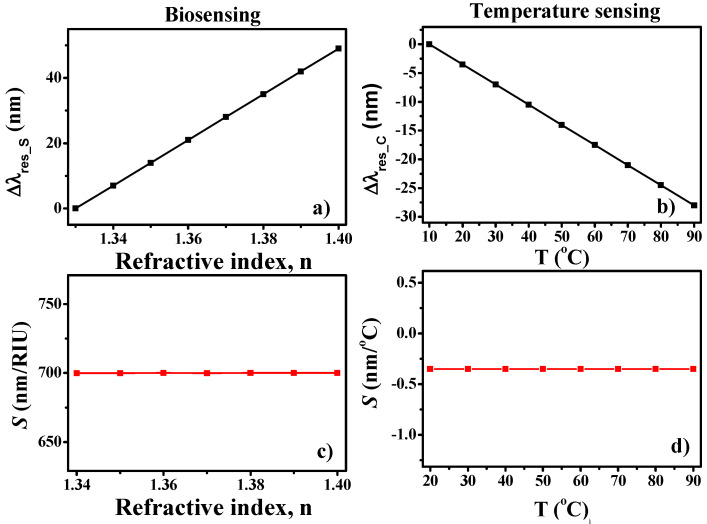
Spectral characteristics: (**a**) ∆λ_res_S_ versus refractive index of the analytes, (**b**) ∆λ_res_C_ versus temperature, (**c**) sensitivity versus refractive index of the analytes, and (**d**) sensitivity versus temperature.

To verify the sensor performance, two important factors should be considered, namely the sensitivity and the figure of merit. The sensitivity (*S*) of the dual-purpose device can be calculated using the following expressions.

(6) $$S_{B}=\frac{\Delta \lambda_{res\_S}}{\Delta n};\;S_{T}=\frac{\Delta \lambda_{res\_C}}{\Delta T}$$

where *S_B_* and *S_T_* are the sensitivity of biosensor and temperature sensor, respectively. ∆*λ_res_S_*, ∆*λ_res_C_*, ∆*n*, and ∆*T* are the change in the resonance wavelength of a square cavity, change in the resonance wavelength of a circular cavity, change in the refractive index, and temperature change, respectively. Figure 9a,b shows the ∆*λ_res_S_* and ∆*λ_res_C_* plot versus RIU and T (°C), respectively. In the case of biosensors, ∆*λ_res_S_* shows an upward linear trend with an increase in RIU. The effective refractive index of the SPP mode increases with an increase in the local refractive index which leads to the shift of λ*_res_S_* to a higher wavelength. However, ∆*λ_res_C_* shows a downward linear trend with an increase in T (°C). This is owing to the reduction in the refractive index of the PDMS material under the influence of increasing temperature. *S_B_* and *S_T_* are determined at 700 nm/RIU and −0.35 nm/°C, respectively, as shown in Figure 9c,d. The sensitivities acquired in the proposed sensor design are quite promising for such simple cavities and dual functionality.

The ability of a plasmonic sensor to quantify slight changes in the refractive index or temperature is directly proportional to *S* and, additionally, inversely proportional to the width of the resonant feature (spectral dip) being tracked. The combination of these parameters is often referred to as the figure of merit (*FOM*) and can be calculated as

*FOM* = *S_B_/*FWHM and *FOM* = *S_T_*/FWHM, (7)

where FWHM is the full width at half maximum of the respective resonance dip. The *FOM* for the biosensing module and temperature sensing module is around 21.9 and 0.008, respectively.

## 5. Fabrication Inaccuracies Influence Analysis

Based on the currently available technology, it is possible to fabricate the nanostructure plasmonic sensors with the fabrication errors of ±5 to ±10 nm [39]. However, inaccuracies may appear during chemical etching of the MIM waveguide patterns owing to careless handling of the etchant. In this section, two different cases of fabrication inaccuracies, i.e., under-etching and over-etching, which may be likely to appear, have been discussed. From this analysis, it will be clear that tolerant is the proposed sensor design against fabrication errors.

### 5.1. Under-Etching Influence

**Figure 10:**
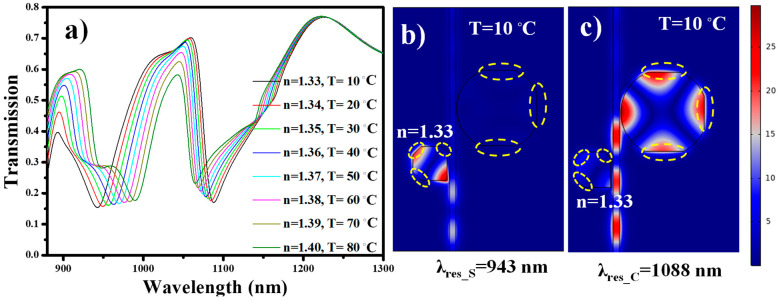
(**a**) Transmission spectrum of the over-etched device employed as biosensor and temperature sensor, (**b**) H-field distribution at λ_res_S_ = 943 nm, and (**c**) H-field distribution at λ_res_C_ = 1088 nm. The yellow dotted circle represents the fabrication inaccuracies.

This situation occurs when the sample is etched for less time than it requires to fully remove the metal layer from the patterned cavity design. There can be different patterns in which inaccuracies can occur. However, here, we have considered three different points-of-inaccuracies for square cavity and three for the circular cavity. The three sides of the cavities are under-etched as a result (~30–40 nm), and the shape is not fully squared and/or circular. This means that the effective side length is smaller than the expected one. The transmission spectrum of the under-etched sample is simulated and plotted in Figure 10a. It can be seen that λ_res_S_ and λ_res_C_ have shifted to a lower wavelength compared with the ones obtained in Figure 7c. There is no variation in *S_B_* and *S_T_*; however, the line shape has deteriorated, which results in lower *FOM*, i.e., 14.58 for biosensing module and 0.0088 for temperature sensing module. The H-field distribution for under-etched square cavity and circular cavity at λ_res_S_ = 943 nm and λ_res_C_ = 1088 nm is mapped as shown in Figure 10b,c respectively.

### 5.2. Over-Etching

**Figure 11:**
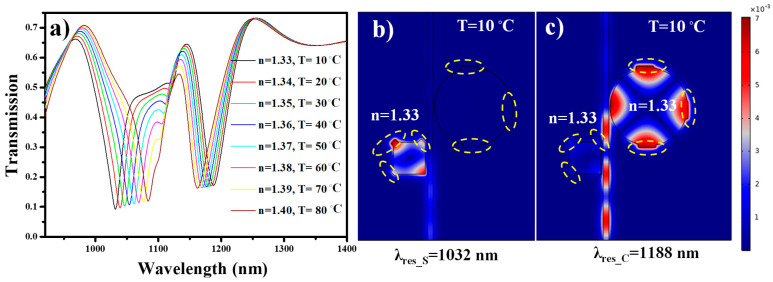
(**a**) Transmission spectrum of the under-etched device employed as biosensor and temperature sensor, (**b**) H-field distribution at λ_res_S_ = 1032 nm, and (**c**) H-field distribution at λ_res_C_ = 1188 nm. The yellow dotted circle represents the fabrication inaccuracies.

Here, the effect of over-etching of the cavities on the device performance was discussed. Like Section 5.1, three points-of-inaccuracies were considered at the same point; however, this time with over-etched patterns. The cavities are not a perfect shape, as was expected. This kind of situation appears when the sample is etched for a longer time than required. The transmission spectrum of the over-etched sample is simulated and plotted in Figure 11a. As the size of the cavities is larger than expected, λ_res_S_ and λ_res_C_ are shifted to higher wavelengths compared with those presented in Figure 7c. *S_B_* and *S_T_* are not influenced and demonstrate a stable value at 700 nm/RIU and −0.35 nm/°C for biosensing module and temperature sensing module, respectively. In this case, the line shape is less effected, which results in a little variation in the *FOM*. The resulting *FOM* of the biosensing and temperature sensing module is around 18.91 and 0.0045, respectively. The H-field distribution for over-etched square cavity and circular cavity at λ_res_S_ = 1032 nm and λ_res_C_ =1188 nm is shown in Figure 11b,c, respectively.

## 6. Limiting Factors

Although the proposed sensor design is unique and multifunctional, it has some preventive factors that should be well-thought-out. When the sensor is employed for a single application (biosensing or temperature sensing), there will be no trouble. However, the dilemma may occur when the device is employed for the simultaneous detection of analytes and temperature. The sensor must not be exposed to extremely high or low temperatures while biosensing [44]. A high temperature can melt or damage the analytes, while a low temperature can freeze them, which can potentially cause inaccuracy in the measurement. For instance, heating a DNA solution above room temperature results in the separation of strands [45]. The temperature when half of the DNA molecule is denatured is termed as the melting temperature, which is around 50–100 °C. Moreover, the melting temperature also depends on several other factors like the length of the DNA and nucleotide sequence composition.

For optimum performance, a temperature-resistant instrumental design is required, which includes temperature control of the light source, temperature control of the analytes, and the use of low-thermal-expansion-coefficient materials for all-optical components. This will be combined with an optical alignment/mechanical arrangement of everything, including the light source, lenses, prism, optical chip, chip holder, and camera, all of which will be insensitive to thermo-mechanical displacements. Moreover, reference sensor cavities can also be employed, which should be maintained at a fixed temperature and refractive index assisting in the calibration of the sensing device.

## 7. Conclusions

Herein, a plasmonic sensor based on an MIM waveguide is proposed for the simultaneous detection of biological analytes and ambient temperature. The sensor is comprised of a square and circular cavity of different effective lengths side coupled to a bus waveguide. As a result, two distinct resonance dips are obtained in the transmission spectrum. The square cavity is filled with a dielectric material such as water (*n* = 1.33) and different analytes are introduced into the cavity, which brings a redshift in the resonance wavelength, whereas the circular cavity is deposited with a thermally sensitive polymer material such as PDMS. Thanks to the high thermo-optic coefficient of the PDMS, the resonance dip related to the circular cavity performs a significant blueshift. As a result, two different functionalities are performed on the same chip independently. The bio-sensitivity and temperature sensitivity are obtained at 700 nm/RIU and −0.35 nm/°C, respectively. The *FOM* for the biosensing module and temperature sensing module is around 21.9 and 0.008, respectively. The proposed sensor design is useful for lab-on-chip diagnostics of the bio analytes in a controlled temperature environment.
